# Phenolic Content and Anti-Inflammatory Activity of Cultivated and Wild-Type *Galium odoratum* Extracts in Murine Bone Marrow-Derived Macrophages

**DOI:** 10.3390/antiox13121447

**Published:** 2024-11-25

**Authors:** Valerija Razzivina, Anna Vasiljeva, Arta Kronberga, Gundars Skudrins, Ilva Nakurte, Marta Zogota, Ieva Mezaka, Osvalds Pugovics, Solveiga Grinberga, Maija Dambrova, Inga Sile

**Affiliations:** 1 Latvian Institute of Organic Synthesis, 21 Aizkraukles Str., LV-1006 Riga, Latvia; valerija@osi.lv (V.R.); maija.dambrova@farm.osi.lv (M.D.); 2 Department of Pharmaceutical Chemistry, Riga Stradins University, 16 Dzirciema Str., LV-1007 Riga, Latvia; 3 Department of Applied Pharmacy, Riga Stradins University, 16 Dzirciema Str., LV-1007 Riga, Latvia; 4 Field and Forest, SIA, 2 Izstades Str., Priekuli Parish, LV-4126 Cēsis County, Latvia; 5 Institute for Environmental Solutions, “Lidlauks”, Priekuli Parish, LV-4126 Cēsis County, Latvia; 6 Laboratory of Finished Dosage Forms, Riga Stradins University, 21 Konsula Str., LV-1007 Riga, Latvia

**Keywords:** *Galium odoratum*, phytochemical composition, coumarin, LC-MS analysis, essential oils, GC-MS analysis, pro-inflammatory activity

## Abstract

*Galium odoratum*, commonly known as sweet woodruff, is a perennial herbaceous plant that contains coumarin and is recognized for its medicinal properties. In this study, the influence of sunlight exposure on the phytochemical composition and anti-inflammatory potential of *G. odoratum* extracts is assessed. The extracts from cultivated and wild-grown plants were analyzed via chromatographic and mass spectrometric methods. In addition, the total phenolic content, antioxidant activity, and effects on macrophage polarization were assessed. The results revealed that while coumarin levels remain stable regardless of environmental conditions, phenolic content and antioxidant activity increase significantly under sun-grown conditions, with chlorogenic acid and rutin identified as major contributing compounds. Additionally, the extracts exhibited anti-inflammatory activity, effectively reducing the M1 macrophage population involved in inflammatory responses. These findings suggest that controlled sunlight exposure can enhance the bioactive profile of *G. odoratum*. This research highlights the critical role of environmental management in optimizing the medicinal properties of *G. odoratum*, providing a foundation for its future use in natural therapeutic applications.

## 1. Introduction

The demand for organically grown medicinal plants is rapidly increasing today due to concerns about environmental pollution associated with conventional agricultural practices, such as the use of chemical pesticides, herbicides, and synthetic fertilizers. Organic farming methods are seen as a more sustainable alternative, as they reduce the negative environmental impacts often caused by these chemicals, including soil degradation, water contamination, and loss of biodiversity. This is especially important in the context of medicinal plant cultivation, where the quality and purity of the plants are crucial to ensuring their efficacy in traditional and modern medicine [1,2].

*Galium odoratum* (L.) Scop. (syn. *Asperula odorata* L.), or sweet woodruff, is an herb of the Rubiaceae family, which is natively distributed throughout Eurasia and northern Africa and was introduced in some locations in the USA (Colorado, Illinois, Minnesota, New York, Vermont) [3]. *G. odoratum* is a perennial herbaceous plant that grows between 10 and 35 cm in height and has an erect, four-angled stem. The lower leaves are arranged in false whorls of five to seven leaves, whereas the upper leaves are arranged in whorls of eight, narrowing at the base. The flowers are white and form spike-like inflorescences at the top of the stem, but they can also be found in the axils of the leaves in the lower whorls. In Latvia, *G. odoratum* typically flowers in May and June [4]. The plant grows best in partial to full shade in moist, rich soils. The plant leaves are notable for their strong fragrance, which resembles the scent of freshly mown hay when the foliage is either crushed or cut. This characteristic sweet odor is due to the presence of coumarin, a natural compound derived from melilotoside, during the drying process [5]. In addition to the main component, coumarin, the chemical composition of *G. odoratum* includes iridoid monoterpene glycosides such as asperuloside, monoterpenes, tannins, iridoids, anthraquinones, flavonoids, and nicotinic acid [6]. These compounds are distributed across different parts of the plant: alizarin and anthracene derivatives are predominantly found in the roots and rhizomes, whereas the aerial parts contain essential oils [7,8], phenolic acids, additional coumarins, flavonoids, tannins, and saponins [9].

Recent research has emphasized the importance of the plant’s population origin and growth conditions in influencing its chemical composition [10]. Environmental factors, such as soil type, climate, and altitude, can significantly affect the concentration of bioactive compounds such as polyphenols and iridoids, which directly impact the biological efficacy of plants. Studies by Ledoux et al. [10] comparing in situ (wild-grown) and ex situ (controlled) populations have demonstrated variations in secondary metabolites, particularly in terms of reduced metabolite content under controlled growth conditions.

Research by Møller et al. [11] revealed that *G. odoratum* grows best in shaded environments, similar to its native deciduous woodlands, where it benefits from dappled light. Under these conditions, the plant maintains high levels of intraindividual variation, particularly in characteristics such as leaf length and width, which are crucial for photosynthesis and overall plant health. Møller et al. [11,12] reported that *G. odoratum* can tolerate various light conditions but shows the best performance under partial to full shade. While plants are moderately adaptable to increased sunlight, excessive exposure can lead to stress, reducing chlorophyll levels and slowing growth. These findings suggest that *G. odoratum* thrives best in environments that closely mimic its natural shaded habitat, ensuring balanced growth and preserving its phenotypic plasticity.

The aerial parts and flowering tops of *G. odoratum* have traditionally been used in herbal medicine for various therapeutic purposes, including the treatment of nervous agitation, jaundice, hemorrhoids, and circulatory and venous disorders. When crushed, plant leaves are used topically to reduce swelling and promote the wound-healing process [6]. Moreover, in Latvian folk medicine, *G. odoratum* is commonly used as a tea and tincture to alleviate gastrointestinal issues such as diarrhea and stomach pain [13]. There have only been a few studies on the biological activity of *G. odoratum* [6,14]. However, the chemical compositions of wild-grown and cultivated *G. odoratum* extracts have not been compared to date. In addition, we studied the antioxidant properties and effects of the extracts on macrophage polarization toward the pro- (M1) and anti-inflammatory (M2) phenotypes.

## 2. Materials and Methods

### 2.1. Plant Materials and Preparation of Extracts

The aerial parts (pooled samples of 20 individuals) of *G. odoratum* from four different habitats were collected in the wild during the flowering stage in May 2019. The collection sites of the wild accessions are detailed in Table S1. Voucher samples were deposited at the Institute for Environmental Solutions (IES) in Latvia with the codes GAL01, GAL03, GAL05, and GAL06.

Rhizomes collected from wild accessions in May 2019 were planted in an organically certified experimental field of IES (57°19′11.7” N 25°19′18.8” E, 115 m altitude). For each accession, a total of 10 plants were planted with a spacing of 30 × 30 cm. Two planting locations were used: an open field (without shading) and an artificial shading system with a 75% shading intensity. The collected samples demonstrated various levels of adaptability to cultivation. Among all the collected accessions, most showed slow development under both open-field cultivation and shaded cultivation; therefore, sample collection for chemical analyses in 2020 was not possible for accessions GAL03 and GAL06, and only accessions GAL01 and GAL05 were collected.

When assessing the appropriateness of the collected *G. odoratum* samples for cultivation, GAL01 (Glāžšķūnis) had the highest plant height (26.3 cm) and was the only accession among those tested to be favorable for mechanized harvesting. Furthermore, this sample demonstrated robust growth and development, resulting in the largest production of fresh herbs per square meter. Consequently, GAL01 (Glāžšķūnis) received the highest rating and was the only sample considered appropriate for cultivation. Therefore, in 2023, a single sample was collected only for chemical analysis.

The collected plant material was dried at 50 °C for 10–16 h and ground into a fine powder. The powdered *G. odoratum* samples were macerated in a 70% ethanol solution (water/ethanol) at a 1:10 *w*/*v* ratio. The mixtures were incubated in the dark for 7 days with periodic cooling and shaking. Afterward, the solutions were clarified through decantation and centrifugation. The resulting extracts were concentrated using a rotary evaporator and then lyophilized into powder form. The final powder was labeled and stored at −20 °C in a refrigerator until further analysis.

In vitro and ex vivo experiments with *G. odoratum* extracts were conducted using lyophilized plant material, which was reconstituted in distilled water.

### 2.2. Chemicals and Reagents

The ethanol (96%) used for extraction was purchased from Kalsnavas Elevators Ltd. (Jaunkalsnava, Latvia). All flavonoid reference compounds and p-coumaric acid were purchased from PhytoLab (Vestenbergsgreuth, Germany). The reference compounds chlorogenic acid and coumarin were obtained from Sigma–Aldrich (Schnelldorf, Germany) and Acros Organics (Geel, Belgium), respectively. Water for UHPLC analysis was purified via a Milli–Q Plus system (Merck Millipore, Burlington, MA, USA), and LiChroslov hypergrade acetonitrile and formic acid were acquired from Sigma–Aldrich (Schnelldorf, Germany). Xylene was purchased from Biochem Chemopharma (Cosne-Cours-sur-Loire, France), and cyclohexane and anhydrous sodium sulfate (Na_2_SO_4_) were purchased from Acros Organics (Geel, Belgium). MTT (3-(4,5-dimethylthiazol-2-yl)-2,5-diphenyltetrazolium bromide), DPPH (2,2-Diphenyl-1-picrylhydrazyl), Folin-Ciocalteu reagent, sodium carbonate (Na_2_CO_3_), gallic acid, L-ascorbic acid, fetal bovine serum (FBS), Hank’s buffered saline solution (HBSS), trypsin, and lipopolysaccharide (LPS), were all sourced from Sigma Aldrich. RPMI-1640 medium with Glutamax was provided by Gibco. Mouse monocyte-colony stimulating factor (M-CSF) and interferon-gamma (IFN-γ) were supplied by Pepro-Tech (London, UK). Antibodies, including FITC-conjugated anti-mouse F4/80, phycoerythrin (PE)-conjugated anti-mouse CD86, and biotin-conjugated anti-mouse CD80, were obtained from BioLegend (San Diego, CA, USA).

### 2.3. UHPLC-HRMS/MS Analysis

The profiling of *G. odoratum* extracts was carried out on a Shimadzu LC–MS hybrid IT–TOF mass spectrometer attached to a Nexera X2 UHPLC system. The column was an Acquity UPLC BEH C18 column (2.1 × 150 mm, 1.7-µm particle size). The separation was performed in gradient mode with eluents A (0.1% formic acid in water) and B (acetonitrile) at a flow rate of 0.4 mL/min. The gradient was as follows: 2% B, 1 min—2% B, 4 min—5% B, 14 min—15% B, 36 min—50% B, 48 min—98% B, 55 min—98% B, 58 min—2% B, and 60 min—2% B. The autosampler was kept at 10 °C, the column oven was kept at 30 °C, and the injection volume was 1 µL.

Electrospray ionization in positive and negative ionization modes was applied, mass range (*m*/*z*)—from 120 to 1000, nebulizing gas (N_2_) flow—1.5 mL/min, CDL temperature—250 °C, detector voltage—1.5 kV, ion accumulation time—100 ms, collision gas—argon and collision energy—50%. Shimadzu LabSolutions Version 3.81.418 software was used for LC–MS data processing. A PDA detector was used to acquire UV–Vis spectra over the range from 210 nm to 800 nm.

Aqueous ethanol extracts of *G. odoratum* were subjected to analysis without initial processing.

### 2.4. UHPLC-MS/MS Analysis

Quantitative analysis of flavonoid glycosides, coumarin, p-coumaric acid, and chlorogenic acid was carried out on a Xevo TQ-S micro (Waters) tandem mass spectrometer operated in positive electrospray ionization mode for all analytes except p-coumaric acid and chlorogenic acid (operated in negative electrospray ionization mode) and multiple reaction monitoring (MRM) mode. The MRM parameters of each analyte were optimized by direct infusion and are shown in Supplementary Materials Table S2. Chromatographic separation was performed on an Acquity BEH C18 column (2.1 × 100 mm, 1.7 µm, Waters) via a Waters Acquity UPLC system. The separation was performed under gradient mode with eluents A (0.1% formic acid in water) and B (acetonitrile) at a flow rate of 0.4 mL/min. The gradient was as follows: 5% B, 0.5 min—5% B, 8 min—98% B, 10 min—98% B, 11 min—5% B, and 12 min—5% B. The column oven was kept at 30 °C, the autosampler was kept at 10 °C, and the injection volume was 1 µL.

Aqueous ethanol extracts of *G. odoratum* were diluted 10 or 100 times with 70% ethanol before quantitative analyses. The target compound concentrations in the extracts were calculated via calibration curves (over a range from 50 ng/mL to 30 µg/mL for all analytes).

### 2.5. Extraction of Essential Oil and Analysis

The essential oils (EOs) from the dried aerial parts of selected *G. odoratum* samples (GAL01, GAL03, GAL05, and GAL06) were extracted via hydrodistillation using a Clevenger-type apparatus for three hours. Fifteen grams of freshly powdered plant material were placed in a 500-mL flask, combined with water, and half a milliliter of xylene was added to the graduated tube to collect the oil. The distillation proceeded at a flow rate of three to four milliliters per minute. After distillation, the organic layer containing the essential oils was isolated, dried using anhydrous sodium sulfate to remove moisture, and stored in sealed vials at 4 °C for subsequent GC-MS analysis. A gas chromatograph (Agilent 7820A) coupled with a mass-selective detector (Agilent 5977B) from Agilent Technologies GmbH, Waldbronn, Germany, was used to analyze the obtained essential oils. The samples were prepared by diluting 100 µL of EO with 900 µL of cyclohexane. Chromatographic separation was achieved using a nonpolar HP-5 capillary column (60 m × 0.25 mm, 0.25-µm film thickness). Helium was used as the carrier gas, maintaining a flow rate of one and a half milliliters per minute with a split ratio of 1:50. The injection volume was 1 μL. The inlet temperature was set at 270 °C, while the oven temperature was methodically increased from 60 °C—held for three minutes—to 290 °C at a rate of 10 °C per minute, with the injector temperature maintained at 280 °C. Mass spectra were obtained at 70 eV, covering a mass-to-charge ratio (*m*/*z*) from 30 to 550, with the ion source temperature fixed at 230 °C. Compounds were identified by comparing retention indices, based on a C5–C24 n-alkane series, with mass spectra from the NIST MS search 2.2 library. GC-MS data analysis was performed using Agilent MassHunter Qualitative and Quantitative Navigator B.08.00. The relative amounts of volatile compounds were determined using peak area normalization.

### 2.6. Determination of the Total Phenolic Content

The total phenolic content (TPC) of *G. odoratum* extracts was determined using the Folin–Ciocalteu colorimetric assay, following the method described by Kähkönen et al. [15], with slight modifications. In summary, 20 µL of the extract was added to a 96-well plate and mixed with 100 µL of 10% Folin–Ciocalteu reagent. Then, 80 µL of a 7.5% Na_2_CO_3_ solution was added to the mixture. The plate was incubated in the dark at room temperature for 30 min with gentle shaking. After incubation, absorbance was measured at 765 nm using a Hidex Sense microplate reader. A calibration curve using gallic acid as the standard was prepared, and TPC was expressed as milligrams of gallic acid equivalent (GAE) per gram of lyophilized extract. All experiments were performed in triplicate.

### 2.7. 2,2-Diphenyl-1-picrylhydrazyl (DPPH) Free Radical Scavenging Assay

The antioxidant activity of *G. odoratum* extracts was assessed using the DPPH free radical scavenging assay, based on the method described by Brand-Williams et al. [16], with minor modifications. In this assay, 20 μL of the water/diluted extract was mixed with 180 μL of DPPH solution (40 μg/mL in methanol) in a 96-well plate. The plate was incubated at room temperature in the dark for 15 min. After incubation, the absorbance was measured at 517 nm using a Hidex Sense microplate reader to determine the reduction in DPPH free radicals. Ascorbic acid solutions, ranging from 0 to 800 μg/mL, served as the standard. Various concentrations of the lyophilized extract, dissolved in water, were tested to determine the IC50, which is the concentration needed to reduce the DPPH absorbance by 50%. The radical scavenging activity was calculated via the following formula:

DPPH radical scavenging activity (%) = [(A0 − A1)/A0] × 100,

where A0 represents the absorbance of the control and A1 represents the absorbance of the sample.

### 2.8. Isolation of Bone Marrow-Derived Macrophages

Bone marrow-derived macrophages (BMDMs) were obtained from male C57BL6/J inbred mice (18–20 weeks old; Envigo, The Netherlands) following previously established protocols, with slight modifications [17,18]. The cells were cultured for 6–7 days in RPMI-1640 medium containing GlutaMAX, 10% FBS, 1% antibiotics (penicillin at 100 U/mL and streptomycin at 100 μg/mL), and 10 ng/mL M-CSF. All experimental procedures complied with European Community guidelines (2010/63/EU), local laws, and policies and were approved by the Latvian Animal Protection Ethical Committee, Food and Veterinary Service, Riga, Latvia.

BMDMs in Petri dishes were washed twice with HBSS, followed by detachment using 0.5% trypsin. The detached cells were transferred to RPMI-1640 medium containing 10% FBS and 1% antibiotics, then centrifuged at 300× *g* for 5 min at room temperature. After centrifugation, the cells were resuspended in RPMI-1640 medium supplemented with 10% FBS and 1% antibiotics and seeded into 12-well or 96-well plates. The cells were then incubated at 37 °C in a HERAcell VIOS 160i CO_2_ incubator (Thermo Fisher Scientific, Waltham, MA, USA) with 5% CO_2_ for at least 1 h prior to the MTT assay, polarization toward the M1 and M2 phenotypes, and analysis by flow cytometry.

### 2.9. Assessment of Cell Viability Using the MTT Assay

The MTT assay was used to evaluate the viability of BMDMs after 24 h of incubation with varying concentrations of *G. odoratum* extract. The BMDMs were plated in 96-well plates at a final density of 1.2 × 10^6^ cells/mL. The *G. odoratum* extract was dissolved in water, and final concentrations ranging from 50 µg/mL to 750 µg/mL were prepared by diluting the extract in the culture medium. Control cells were treated with water alone, without the extract. After incubation with the extract, the cells were exposed to an MTT solution (1 mg/mL) and incubated at 37 °C for 1–2 h. Following incubation, the medium was carefully removed, and isopropanol was added to each well to solubilize the formazan crystals produced. Absorbance was then measured at 570 nm, with a reference wavelength of 650 nm, using a Hidex Sense microplate reader. The percentage of viable cells was calculated using the formula:

(%) = [100 × (sample abs)/(control abs)].

### 2.10. Treatment of Bone Marrow-Derived Macrophages with the Extract, Polarization Toward the M1 and M2 Phenotypes, and Assessment via Flow Cytometry

BMDMs were plated in 12-well plates at a concentration of 6.5 × 10^5^ cells/mL. To promote polarization toward the M1 (proinflammatory) phenotype, the cells were stimulated with 5 ng/mL LPS and 10 U/mL murine IFN-γ or with IL-4 (interleukin-4) at a concentration of 10 ng/mL to induce polarization toward the M2 (anti-inflammatory) phenotype. Both treatments were conducted in the presence of GAL05 extracts (250 µg/mL and 500 µg/mL) for 24 h.

Following incubation, the cells were washed twice with HBSS and detached using 0.5% trypsin. The detached cells were resuspended in RPMI medium containing 10% FBS and 1% antibiotic, then centrifuged at 300× *g* for 5 min. The cell suspension was then incubated with specific antibody mixtures (1:200 dilution) for 45 min on ice in the dark. For M1-polarized cells, the antibody panel included FITC-conjugated anti-mouse F4/80, phycoerythrin (PE)-conjugated anti-mouse CD86, and biotin-conjugated anti-mouse CD80. For M2-polarized cells, the antibody panel consisted of FITC-conjugated anti-mouse F4/80, PE-conjugated anti-mouse CD206, and PE/Cy7-conjugated anti-mouse CD301. Marker expression was subsequently analyzed using flow cytometry (BD FACSMelody™, BD Biosciences, San Jose, CA, USA). The percentage of the macrophage population was calculated using the following formula:

(%) = [100 × (Number of M1 or M2 Positive Cells)/(Total Number of Cells)].

## 3. Results and Discussion

### 3.1. Qualitative Analysis of G. odoratum

The secondary metabolites of the 70% ethanol extracts of *G. odoratum* were identified via UHPLC-HRMS/MS with an IT-TOF mass analyzer and DAD detector. Representative chromatograms of sun- and shade-grown *G. odoratum* extracts are shown in the Supplementary Materials, Figures S1 and S2. Identification was based on the information provided by the combination of high-resolution mass spectra, fragmentation, analysis of available reference substances, and literature data. Thus, we identified iridoid glucosides (peaks 1, 5, and 8), chlorogenic acid and its isomers (peaks 2–4), four flavonoid glycosides (peaks 6, 7, 9, and 11), and coumarin (peak 10), and the results are summarized in Table 1.

### 3.2. Quantitative Analysis of G. odoratum

The contents of nine major polyphenolic compounds (quercetin 3-rutinoside-7-glucoside, rutin, kaempferol 3,4′-diglucoside-7-rhamnoside, kaempferol 3-rutinoside, coumarin, p-coumaric acid, chlorogenic acid, and two chlorogenic acid isomers) were analyzed in extracts of wild and cultivated (sun-grown and shade-grown) *G. odoratum* (see Table S3). These results indicate that the polyphenolic compound content in *G. odoratum* plants is influenced by growth conditions (wild, cultivated, sun-grown, and shade-grown), plant accession, and season. Compared with those of the accessions GAL01, GAL05, and GAL06, the coumarin content was significantly lower in the extracts prepared from accession GAL03, which represent wild and cultivated sun- and shade-grown plants. In turn, the content of quercetin 3-rutinoside-7-glucoside was greater in the GAL03 accession, which included both sun- and shade-grown conditions. The chlorogenic acid content in the GAL06 accession cultivated under both sun- and shade-grown conditions was lower than that in the GAL01, GAL03, and GAL05 accessions.

The flavonoid content was significantly greater in sun-grown plants than in shade-grown plants harvested in May and June 2021. The content of quercetin 3-rutinoside-7-glucoside in sun-grown plants was 4175 ± 1998 µg/g, whereas its content in shade-grown plants was significantly lower (*p* = 0.02) (49 ± 30 µg/g). Similarly, the content of rutin was significantly greater (*p* = 0.02) in extracts from sun-grown plants (681 ± 324 µg/g) than in those from shade-grown plants (14 ± 10 µg/g). The content of kaempferol 3,4′-diglucoside-7-rhamnoside was also significantly greater (*p* = 0.05) in sun-grown plants (381 ± 112 µg/g) than in shade-grown plants (42 ± 11 µg/g). Additionally, the content of kaempferol 3-rutinoside was significantly greater (*p* = 0.03) in sun-grown plants (722 ± 389 µg/g) than in shade-grown plants (65 ± 13 µg/g).

The content of chlorogenic acid and its isomers was greater in cultivated plants than in wild plants, with no significant differences observed between sun- and shade-grown plants. In wild plants harvested in May 2019, the content of chlorogenic acid was 7110 ± 1450 µg/g, whereas in cultivated sun- and shade-grown plants harvested in June 2020, the chlorogenic acid content was 19,199 ± 2910 µg/g and 12,491 ± 4073 µg/g, respectively.

Compared with those harvested in 2021, the plants harvested in 2020 presented significantly greater contents of quercetin 3-rutinoside 7-glucoside, rutin, kaempferol 3,4′-diglucoside 7-rhamnoside, and kaempferol 3-rutinoside under both sun- and shade-grown conditions.

**Figure 1:**
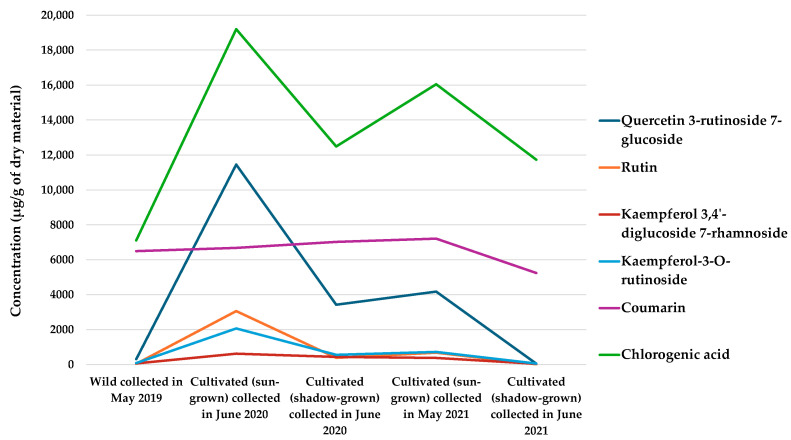
Variation in the phytochemical concentration of aqueous ethanol extracts of wild and cultivated (sun- and shade-grown) *G. odoratum* over time and under different growth conditions.

The cultivation conditions (wild, cultivated—sun-grown, and shade-grown) did not significantly affect the levels of coumarin or p-coumaric acid. However, the content of phenolic compounds varied with harvest year and specific plant accessions (Figure 1).

### 3.3. Extraction of Essential Oils and Analysis

On the basis of previously published findings [8], *G. odoratum* is not typically recognized as a significant essential oil (EO) plant. Our study supports this observation, as the EO yield from all tested samples ranged from 0.35 to 0.69 mL kg^−1^ dry mass (Table 2).

This relatively low yield was consistent across various growing conditions, including plants cultivated in both sunny and shaded environments, as well as in wild and cultivated environments, suggesting notable stability in EO production regardless of external factors. Such stability across different environments highlights the plant’s resilience in terms of EO yield, even though the overall yield remains modest compared with that of more prominent EO-producing species. Nevertheless, the chemical composition of essential oil volatiles has garnered increased scientific interest because of their potential applications and bioactive properties. Using gas chromatography-mass spectrometry (GC-MS) analysis, we identified 20 distinct compounds in the essential oils (Table 3), demonstrating the plants’ chemical diversity.

**Figure 2:**
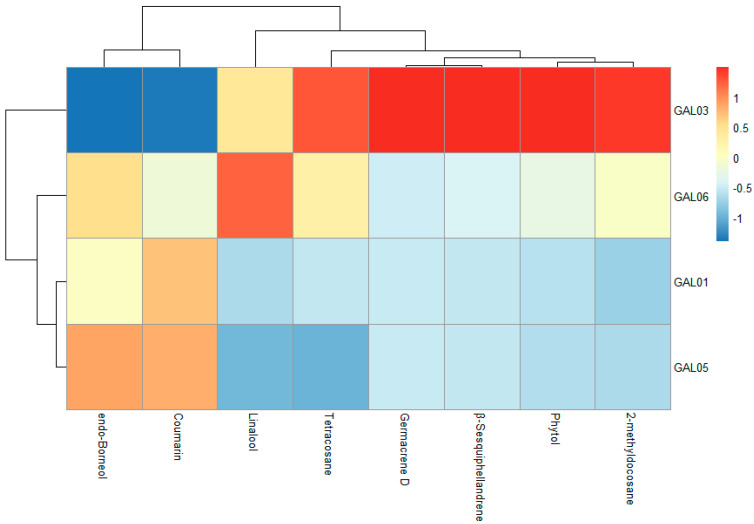
Heatmap depicting average values of dominant chemical components in the essential oils of wild and field-grown *G. odoratum* across various samples.

Significant differences in volatile compounds were observed only between the samples (*p* < 0.05); however, their growing conditions—whether in the shade or sun and whether in the wild or cultivated in a garden—did not have any significant influence. Figure 2 presents the results (mean values from all measurements) of a statistical analysis depicted via a heatmap dendrogram of the eight most dominant compounds. The heatmap utilizes a color gradient where red indicates relatively high relative concentrations, and the spectrum from yellow to blue represents relatively low concentrations of volatile compounds. The identification of these dominant compounds is particularly important, as they may be responsible for the characteristic aroma of *G. odoratum* and its potential biological activities, such as providing a protective effect against oxidative DNA damage in human lymphocyte cells [19].

Like the evaluation of nonvolatile compounds, distinct differences in the chemical composition of sample GAL03 were observed compared with those of the other three samples. Specifically, GAL03 presented the lowest concentration of coumarin but significantly higher concentrations of germacrene D, phytol, and tetracosane. These variations suggest that, compared with other samples, GAL03 may possess a unique chemical profile, which could influence its potential applications or biological activities. In the GAL06 sample, alongside the dominant coumarin, there was a significantly high concentration of linalool and tetracosane. The chemical compositions of samples GAL01 and GAL05 were similar, each containing a high concentration of coumarin, exceeding 70%. These observations suggest that although *G. odoratum* is considered a single species, the specific location where the plant grows has a significant influence on its chemical profile. This finding is corroborated by previous studies, such as those conducted by Başer et al. [8], where coumarin was completely absent in *G. odoratum* samples from Turkey. Conversely, an earlier study by Wörner et al. [7] investigating the volatile compound fraction in samples obtained via solid–liquid extraction via a pentane/dichloromethane mixture (2:1, *v*/*v*) from dried woodruff collected in Germany identified coumarin as the dominant compound. Such variations underscore the importance of geographic and environmental factors in determining the chemical composition of plants.

### 3.4. The Total Content of Phenolic Compounds and DPPH Free Radical Scavenging Activity of G. odoratum Extracts

This study compared the total phenolic content (TPC) and DPPH free radical scavenging activity of *G. odoratum* extracts collected in different years from wild-grown plants in 2019 and cultivated plants in 2020 and 2021, depending on whether they were grown in sunlight or shade. The highest TPC result (105.64 ± 4.94 mg GAE/g) was observed in samples collected in June 2020 from plants grown under sunny conditions (Table 4). Similarly, plants cultivated in sunlight in May 2021 and those grown in the shade in June 2021 presented comparable TPC levels (92.27 ± 8.01 mg GAE/g and 92.33 ± 11.15 mg GAE/g, respectively). In contrast, plants cultivated under shade in June 2020 presented a significantly lower TPC (66.23 ± 8.57 mg GAE/g), which was even lower than that of wild-grown *G. odoratum* plants collected in May 2019.

The cultivated *G. odoratum* plants grown in sunlight in June 2020 presented the highest antioxidant activity (DPPH) among the samples, as evidenced by the lowest average IC50 value of 529 ± 17 µg/mL. These findings suggest that sunlight exposure during cultivation significantly enhances the antioxidant properties of *G. odoratum*, possibly through increased synthesis of phenolic compounds or other bioactive constituents that contribute to its radical scavenging activity. Conversely, the wild plants collected in May 2019 and the shade-grown plants collected in June 2020 presented the weakest antioxidant activities. These findings indicate that environmental factors such as natural habitat conditions in the wild and limited sunlight exposure during cultivation can adversely affect the antioxidant potency of plants.

Overall, these results highlight the significant influence of sunlight on both the TPC and the antioxidant capacity of *G. odoratum*. Plants grown under sunny conditions consistently presented higher TPC values, which were correlated with lower IC50 values in the DPPH assay, indicating stronger antioxidant activity. These findings suggest that controlled cultivation, particularly with adequate sunlight exposure, not only enhances the accumulation of phenolic compounds but also optimizes the overall antioxidant potential of *G. odoratum*. Consequently, these findings underscore the importance of sunlight in maximizing the plant’s bioactive properties, which could be beneficial for its use in various therapeutic applications.

A comparison of our *G. odoratum* DPPH results with those from Vlase et al. [20] revealed that our IC50 values (529 ± 17 µg/mL to 1005 ± 131 µg/mL) are greater, indicating weaker antioxidant activity than the reported value of 264.42 ± 0.74 µg/mL. This difference may result from variations in extraction methods, geographical origins, and environmental factors, such as sunlight exposure, which significantly influence the antioxidant potential. Our findings emphasize the importance of cultivation conditions, particularly sunlight, in enhancing the antioxidant properties of *G. odoratum*.

Furthermore, our study revealed that the IC50 values of *G. odoratum* in the DPPH assay ranged from 529 ± 17 µg/mL to 1005 ± 131 µg/mL, depending on the cultivation conditions, with sunlight exposure enhancing antioxidant activity. In contrast, the antioxidant potential of *G. odoratum* in the study by Hanganu et al. [21] was shown to be strong across different assays (FRAP, CUPRAC, xanthine oxidase inhibition), with their results indicating that significant antioxidant capacity was correlated with high polyphenol content. These findings, together with our results, suggest that environmental factors such as sunlight exposure play crucial roles in maximizing the antioxidant properties of *G. odoratum*, potentially enhancing its therapeutic efficacy.

The aerial parts of wild *G. odoratum* accessions (GAL01 and GAL05) collected from various regions of Latvia were analyzed. The results are expressed as the means ± standard deviations (SDs) from three independent experiments, each conducted in duplicate. The total phenolic content is reported as milligrams of gallic acid equivalents (mg GAE) per gram of lyophilized extract.

### 3.5. The Effects of G. odoratum Extracts on the Polarization of Bone Marrow-Derived Macrophages Toward the M1 and M2 Phenotypes

**Figure 3:**
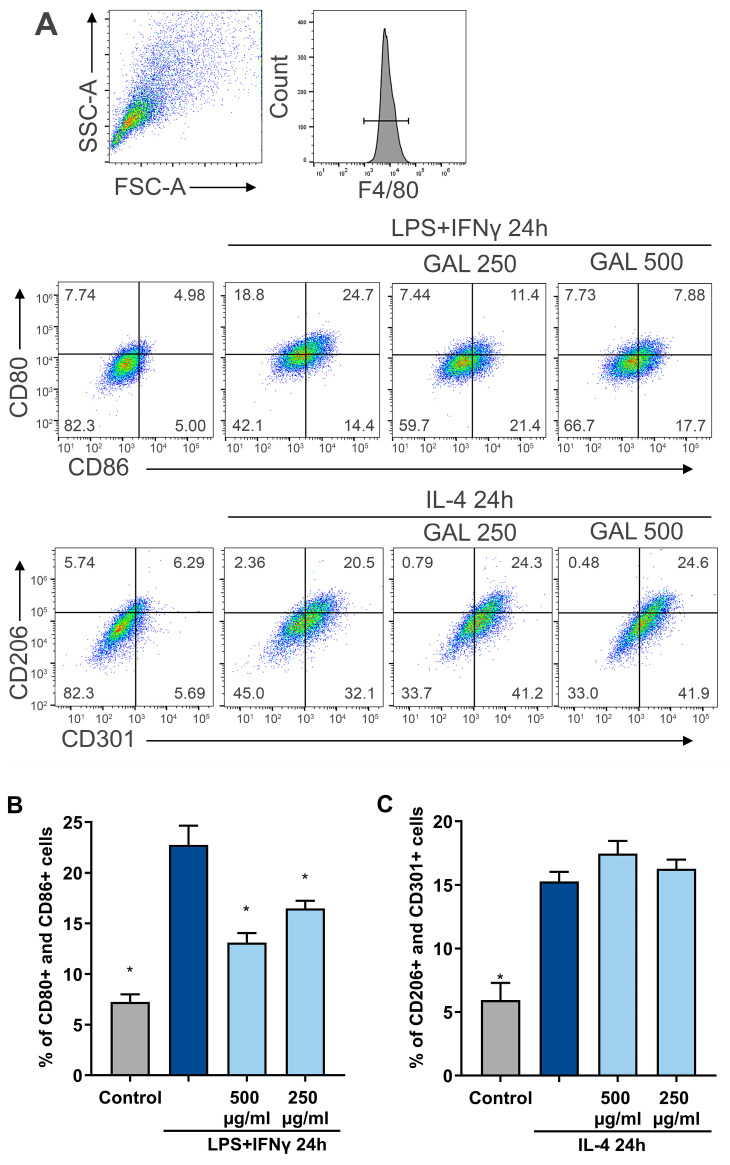
Flow cytometry analysis of bone marrow-derived macrophage (BMDM) polarization toward the M1 and M2 phenotypes. (**A**) In the upper right quadrant, F4/80-positive cells were selected for dual analysis of CD80 and CD86 (representing the M1 macrophage phenotype) and CD206 and CD301 (representing the M2 macrophage phenotype). A dot plot summarizing results from three separate experiments, each conducted in triplicate, is displayed at the bottom of the figure. (**B**) Proinflammatory surface markers CD80 and CD86 were assessed via flow cytometry 24 h post-treatment of BMDMs with the extract at concentrations of 250 µg/mL and 500 µg/mL, as well as with LPS/IFN-γ. (**C**) Anti-inflammatory markers CD206 and CD301 were similarly evaluated by flow cytometry 24 h after treating BMDMs with the extract and IL-4. The extract used in this study was derived from GAL05 flowering shoots grown under sunlight in June 2020. Results are presented as mean values ± SEM, based on three independent measurements from three parallel experiments. Statistical differences were determined using one-way ANOVA followed by Dunnett’s test. * Indicates significant difference compared to the LPS or IL-4 control group, with *p* < 0.05.

In the present study, we investigated the effects of *G. odoratum* extract on macrophage polarization toward the proinflammatory M1 phenotype, which is stimulated by LPS and IFN-γ and characterized by the presence of the surface markers CD80 and CD86. Compared with that of the untreated control, the number of M1-polarized macrophages increased threefold (Figure 3B). Accession GAL05 was selected for further experiments because it showed the most promising results after chemical composition analysis, having the highest amount of total phenolic compounds. The proportion of CD80+ and CD86+ double-positive cells, indicative of M1 macrophages, was 23% following stimulation with LPS/IFN-γ. Treatment with *G. odoratum* extracts at 250 µg/mL and 500 µg/mL significantly decreased the M1 macrophage population to 16% and 13%, respectively, after 24 h. Conversely, the percentage of CD206+ and CD301+ double-positive cells, representing M2 macrophages, was 15% after IL-4 stimulation. Incubation with *G. odoratum* extracts at concentrations of 250 µg/mL and 500 µg/mL increased the M2 macrophage population to 16% and 17%, respectively, over 24 h; however, this increase was not statistically significant (Figure 3C). In our experiments, markers CD86 and CD80 were used for M1-polarized cells, while CD206 and CD301 were used for M2-polarized cells due to their specificity for these populations, as supported by the literature [22,23]. These results indicate that the *G. odoratum* extract significantly reduced the M1 macrophage population but did not substantially affect the polarization of macrophages toward the anti-inflammatory M2 phenotype. The decrease in the M1 population, without a significant M2 increase, suggests that *G. odoratum* extract reduces proinflammatory responses without strongly driving M2 polarization. This may result from its suppression of inflammatory signaling or its role as an immunomodulator, balancing inflammatory and anti-inflammatory states. Further studies are needed to explore whether prolonged treatment, varied doses, or additional stimuli could enhance M2 polarization. The MTT assay results showed that the *G. odoratum* extract exhibited no toxicity toward BMDMs when applied for 24 h at concentrations ranging from 50 to 750 μg/mL (Figure S3).

The *G. odoratum* extracts identified in our study contain compounds such as chlorogenic acid, coumarins, rutin, and asperuloside, which are likely responsible for the total phenolic content, antioxidant activity, and anti-inflammatory effects on M1 macrophages. Chlorogenic acid and coumarin are well known for their potent antioxidant properties and are capable of neutralizing free radicals and reducing oxidative stress [24,25]. Rutin, a flavonoid, is recognized for its ability to stabilize capillaries and reduce inflammation [26], whereas asperuloside has been associated with anti-inflammatory and immunomodulatory effects [27]. The synergistic action of these compounds may explain why our *G. odoratum* extracts exhibit antioxidant activity and effectively reduce M1 macrophage polarization, thereby offering the potential for modulating inflammatory responses. Both *Galium verum* and *Galium odoratum* have previously demonstrated significant anti-inflammatory properties, which aligns with their traditional use in treating skin disorders and promoting wound healing. A study on *G. verum* highlighted its ability to reduce proinflammatory cytokines such as IL-6 and CXCL8, emphasizing its potential as a therapeutic agent for managing inflammatory conditions [28]. Similarly, *G. odoratum* was found to contain bioactive compounds that modulate immune responses by decreasing the production of proinflammatory cytokines, further supporting its application in inflammatory treatments [6]. The complementary findings from our studies highlight the therapeutic value of these *Galium* species in managing inflammation, potentially offering natural alternatives for treating inflammation-related disorders.

## 4. Conclusions

The coumarin content in *G. odoratum* remains relatively stable across various environmental conditions, including variations in sunlight exposure and whether the plants are wild or cultivated. Although the content of phenolic compounds and antioxidant properties in extracts are increased by sunlight and growth conditions, coumarin levels do not significantly fluctuate because of these factors. This stability suggests that the coumarin content in *G. odoratum* is determined primarily by genetic rather than environmental factors. In contrast, the phenolic content and antioxidant activity of *G. odoratum* are notably responsive to environmental influences, particularly sunlight. The cultivated, sun-grown plants presented the highest levels of phenolic compounds, with chlorogenic acid and rutin as key contributors. *G. odoratum* also exerts anti-inflammatory effects by reducing the proportion of proinflammatory M1 macrophages, indicating its potential for the development of natural treatments for inflammation and oxidative damage. Optimizing sunlight exposure may thus enhance the bioactive properties of *G. odoratum*, supporting its medicinal potential.

## Figures and Tables

**Table 1:** Phytocomponents identified in the 70% ethanol extracts of *G. odoratum*.

Peak	RT, min (MS)	Compound	MW	Characteristic Ions, *m*/*z*		Calculated Elemental Composition *
				ESI+	ESI−	
1	6.0	Geniposidic acid	374	397.11 [M + Na]	373.112	C_16_H_22_O_10_
2	6.5	Chlorogenic acid (isomer)	354	377.085 [M + Na]	353.086	C_16_H_18_O_9_
3	9.2	**Chlorogenic acid**	354	355.103	353.086	C_16_H_18_O_9_
4	10.2	Chlorogenic acid (isomer)	354	377.085 [M + Na]	353.086	C_16_H_18_O_9_
5	10.8	Asperuloside	414	437.105 [M + Na], 287.056	459.113 [M + HCOO]	C_18_H_22_O_11_
6	11.5	**Quercetin 3-O-rutinoside-7-O-glucoside**	772	773.207, 465.101, 303.049	771.197	C_33_H_40_O_21_
7	13.1	**Kaempferol 3,4′-diglucoside 7-rhamnoside**	756	757.215, 449.107, 287.053	755.202	C_33_H_40_O_20_
8	14.1	Melilotoside	326	349.089 [M + Na]	325.089	C_15_H_18_O_8_
9	17.1	**Rutin**	610	633.141 [M + Na], 303.049	609.143	C_27_H_30_O_12_
10	17.6	**Coumarin**	146	147.044	-	C_9_H_6_O_2_
11	18.7	**Kaempferol 3-O-rutinoside**	594	595.163	593.331	C_27_H_30_O_15_

* mass difference within ±5 mDa, **bold**—identified by comparison to reference compounds.

**Table 2:** Essential oil (EO) content (mg kg^−1^ DW) of *G. odoratum* in wild and field-grown conditions over different years of vegetation.

Sample Code	Growth Conditions	Collection Time	EO, mL kg^−1^ DW
GAL01		22 May 2019	0.36 ± 0.01
GAL02		31 May 2019	0.35 ± 0.01
GAL03	Wild	29 May 2019	0.35 ± 0.01
GAL06		30 May 2019	0.69 ± 0.02
GAL01	Field grown, first vegetation season, sunlight	5 June 2020	0.35 ± 0.01
GAL03		5 June 2020	0.36 ± 0.01
GAL05		5 June 2020	0.35 ± 0.01
GAL06		5 June 2020	0.60 ± 0.01
GAL01	Field grown, first vegetation season, shade	5 June 2020	0.36 ± 0.01
GAL05		5 June 2020	0.37 ± 0.01
GAL01	Field grown, second vegetation season, sunlight	25 May 2021	0.36 ± 0.01
GAL03		25 May 2021	0.35 ± 0.01
GAL05		25 May 2021	0.35 ± 0.01
GAL06		25 May 2021	0.67 ± 0.02
GAL01	Field grown, second vegetation season, shade	8 June 2021	0.38 ± 0.01
GAL03		8 June 2021	0.37 ± 0.01
GAL05		8 June 2021	0.37 ± 0.01
GAL06		8 June 2021	0.65 ± 0.02

**Table 3:** Composition (%) of volatile compounds in essential oils derived from dried *G. odoratum* plants.

RI ^a^	Compound ^b^	Formula ^b^	Class	Composition Range, %
1079	4-Methylbenzaldehyde	C_8_H_8_O	Aldehydes	0.3–1.21
1099	Linalool	C_10_H_18_O	Terpenoids	3.46–15.44
1167	endo-Borneol	C_10_H_18_O	Terpenoids	2.6–12.32
1242	Carvone	C_10_H_14_O	Terpenoids	n.d–0.27
1291	Thymol	C_10_H_14_O	Terpenoids	n.d–0.41
1384	α-Bourbonene	C_15_H_24_	Sesquiterpenoids	n.d–1.04
1441	Coumarin	C_9_H_6_O_2_	Lactones	24.8–77.46
1481	Germacrene D	C_15_H_24_	Sesquiterpenoids	n.d–15.08
1482	Cadina-1(6),4-diene	C_15_H_24_	Sesquiterpenoids	n.d–0.64
1524	β-Sesquiphellandrene	C_15_H_24_	Sesquiterpenoids	n.d–1.14
2011	Unknown			n.d–0.39
2054	Unknown			n.d–0.09
2114	Phytol	C_20_H_40_O	Diterpene alcohol	2.15–12.0
2119	Unknown			n.d–0.51
2123	Unknown			n.d–0.50
2124	3-Ethyl-3-methylnonadecane	C_22_H_46_	Hydrocarbons	0.16–2.73
2181	Unknown			n.d–0.23
2263	2-Methyldocosane	C_23_H_48_	Hydrocarbons	0.31–3.86
2400	Tetracosane	C_24_H_50_	Hydrocarbons	2.61–18.23
2500	Unknown			0.65–3.38

^a^ Retention indices (RIs) determined from the HP-5MS capillary column. ^b^ Based on the NIST (National Institute of Standards and Technology) MS Search 2.2 library, n.d—not detected.

**Table 4:** Total phenolic content and DPPH free radical scavenging activity of *G. odoratum* extracts from wild-grown and cultivated (grown in sunlight and shade) flowering shoots.

Plant Sample	TPC (mg GAE/g Lyophilized Extract Wt)	IC50 Value of DPPH Radical Scavenging Activity (μg/mL)
**Wild (May 2019)**		
GAL01	80.39	746
GAL05	62.44	1206
Average ± SD	**71.42 ± 11.07**	**976 ± 133**
**Cultivated (sun-grown, June 2020)**		
GAL01	103.60	499
GAL05	107.68	559
Average ± SD	**105.64 ± 4.94**	**529 ± 17**
**Cultivated (shade-grown, June 2020)**		
GAL01	61.97	1232
GAL05	70.50	777
Average ± SD	**66.23 ± 8.57**	**1005 ± 131**
**Cultivated (sun-grown, May 2021)**		
GAL01	87.26	628
GAL05	97.27	586
Average ± SD	**92.27 ± 8.01**	**607 ± 12**
**Cultivated (shade-grown, June 2021)**		
GAL01	82.66	644
GAL05	102.00	505
Average ± SD	**92.33 ± 11.15**	**575 ± 40**
Ascorbic acid		**44 ± 1**

## Data Availability

Data are contained within the article or Supplementary Materials.

## Supplementary Materials

The following supporting information can be downloaded at: https://www.mdpi.com/article/10.3390/antiox13121447/s1: Figure S1: Representative chromatogram of *G. odoratum* (sun-grown) 70% ethanol extract; Figure S2: Representative chromatogram of *G. odoratum* (shade-grown) 70% ethanol extract; Figure S3: Effects of *G. odoratum* extract on bone marrow-derived macrophage viability measured by the MTT assay; Table S1: Growth conditions, collection time and locations of the collected wild and cultivated *G. odoratum* accessions; Table S2: MRM parameters for the analysis of flavonoids, coumarin and phenolic acids in *G. odoratum*; Table S3: Content of flavonoids, coumarin and phenolic acid (µg/g of dry material) in aqueous ethanol extracts of wild and cultivated (sun-grown and shade-grown) *G. odoratum* samples.
