# Supplementary material for: Phenolic Content and Anti-Inflammatory Activity of Cultivated and Wild-Type Galium odoratum Extracts in Murine Bone Marrow-Derived Macrophages
Source: Antioxidants (Basel). 2024 Nov 25;13(12):1447. doi: 10.3390/antiox13121447 (PMC11672921; doi:10.3390/antiox13121447)
Supplement: Supplementary file 1 [file antioxidants-13-01447-s001.zip › antioxidants-3309878-supplementary.pdf]

Supplementary data to:

Phenolic content and anti-inflammatory activity of cultivated and wild-type *Galium odoratum* extracts in murine bone mar-row-derived macrophages

Supplementary Table S1. Growth conditions, collection time and locations of the collected wild and cultivated *G. odoratum* accessions

Accession denomination	Growth conditions	Collection time	Location	Latitude	Longitude	Elevation
GAL01	Wild	22.05.2019	Glāzšķūnis	56°46'54.4"N	24°53'22.4"E	81 m
GAL02		31.05.2019	Amata	57°07'26.0"N	25°26'08.7"E	200 m
GAL03		29.05.2019	Jaunpiebalga	57°08'16.4"N	26°08'06.4"E	210 m
GAL05		29.05.2019	Ērgļi	56°54'04.8"N	24°40'23.1"E	198 m
GAL06		30.05.2019	Staicele	57°54'20.4"N	24°40'23.1"E	49 m
GAL01<br>GAL03<br>GAL05<br>GAL06	Field grown, first vegetation season, sunlight	05.06.2020<br>05.06.2020<br>05.06.2020<br>05.06.2020	IES garden, Priekuļi	57°19'11.7"N	25°19'18.8"E	115 m
GAL01<br>GAL05	Field grown, first vegetation season, shade	05.06.2020<br>05.06.2020				
GAL01<br>GAL03<br>GAL05<br>GAL06	Field grown, second vegetation season, sunlight	25.05.2021<br>25.05.2021<br>25.05.2021<br>25.05.2021				
GAL01<br>GAL03<br>GAL05<br>GAL06	Field grown, second vegetation season, shade	08.06.2021<br>08.06.2021<br>08.06.2021.<br>08.06.2021				
GAL01	Field grown, fourth vegetation season, shade	31.05.2023<br>30.08.2023				

Supplementary Table S2. MRM parameters for the analysis of flavonoids, coumarin, and phenolic acids in *G. odoratum*

Compound	Polarity	MS/MS	Cone, V	Collision energy, eV
Kaempferol-3-O-rutinoside	ESI+	595 > 287	30	15
Kaempferol 3,4'-diglucoside 7-rhamnoside	ESI+	757 > 287	30	20
Quercetin 3-rutinoside 7-glucoside	ESI+	773 > 303	30	20
Rutin	ESI+	611 > 303	30	20
Coumarin	ESI+	147 > 91<br>147 > 103	30	20
Chlorogenic acids	ESI-	353 > 191	20	15
p-coumaric acid	ESI-	163 > 119	15	15

Supplementary Table S3. Content of flavonoids, coumarin, and phenolic acid (µg/g of dry material) in aqueous ethanol extracts of wild and cultivated (sun-grown and shade-grown) *G. odoratum* samples

Sample	Quercetin 3-rutinoside 7-glucoside	Rutin	Kaempferol 3,4'-diglucoside 7-rhamnoside	Kaempferol-3-O-rutinoside	Coumarin	p-coumaric acid	Chlorogenic acid	Chlorogenic acid isomer 1	Chlorogenic acid isomer 2
**Wild collected in May 2019**									
GAL01	178.1	16.9	64.3	65.7	6046.8	4.9	6311.8	434.7	213.2
GAL03	366.0	30.6	26.7	29.7	1474.3	20.1	9619.0	675.7	275.2
GAL05	503.6	98.0	116.9	175.1	8121.4	18.6	6332.3	377.7	125.2
GAL06	182.4	20.9	68.5	86.5	10331.3	5.2	6175.7	520.4	97.7
Average:	**308**	**42**	**69**	**89**	**6493**	**12.2**	**7110**	**502**	**178**
SD:	136	33	32	54	3270	7	1450	112	71
**Cultivated (sun-grown) collected in June 2020**									
GAL01	13127.6	1085.4	657.3	752.9	8056.8	4.8	21180.1	858.1	362.6
GAL03	15339.0	2943.2	368.6	1054.3	1338.7	8.1	19958.8	653.7	446.0
GAL05	9970.4	2733.7	1008.0	2305.8	8159.6	6.0	21407.2	633.6	294.3
GAL06	7350.3	5510.0	487.2	4146.4	9182.8	4.4	14249.5	693.5	280.1
Average:	**11447**	**3068**	**630**	**2065**	**6684**	**5.8**	**19199**	**710**	**346**
SD:	3039	1583	241	1335	3118	1	2910	88	66
**Cultivated (shade-grown) collected in June 2020**									
GAL01	929.5	109.7	152.2	201.5	6461.4	4.6	8418.0	490.7	97.1
GAL05	5914.4	666.0	727.0	913.9	7590.0	7.1	16563.9	763.3	209.9
Average:	**3422**	**388**	**440**	**558**	**7026**	**5.8**	**12491**	**627**	**154**
SD:	2492	278	287	356	564	1	4073	136	56
**Cultivated (sun-grown) collected in May 2021**									
GAL01	4905.4	236.5	495.9	303.0	7541.2	3.8	17218.9	676.6	463.5
GAL03	7054.4	1122.3	292.4	501.9	1749.9	17.0	20593.7	694.5	671.6
GAL05	2934.2	565.3	488.5	744.4	9184.6	11.4	14594.7	569.6	384.3
GAL06	1806.2	798.2	248.5	1338.6	10359.2	5.5	11780.6	531.4	280.5
Average:	**4175**	**681**	**381**	**722**	**7209**	**9.4**	**16047**	**618**	**450**
SD:	1998	324	112	389	3307	5	3254	69	143
**Cultivated (shade-grown) collected in June 2021**									
GAL01	55,5	9.0	55.6	44.9	5123.1	8.6	14978.0	446.2	356.9
GAL03	95,4	32.6	32.6	78.4	1303.9	19.4	13317.6	545.7	397.8
GAL05	21,6	8.1	48.5	71.2	5002.8	6.8	13213.4	489.3	421.2
GAL06	23,2	8.0	31.6	63.7	9531.9	6.2	5413.0	418.6	100.3
Average:	**49**	**14**	**42**	**65**	**5240**	**10.2**	**11731**	**475**	**329**
SD:	30	10	11	13	2915	5	3714	48	128
**Cultivated (shade-grown) collected in May 2023 (before flowering)**									
GAL01	125.1	36.6	466.2	120.9	1782.4	14.6	12014.8		
**Cultivated (shade-grown) collected in May 2023 (flowering)**									
GAL01	57.3	15.6	345.2	85.0	3296.6	10.6	9058.4		
**Cultivated (shade-grown) collected in August 2023 (after flowering)**									
GAL01	70.2	22.6	597.5	282.6	4875.6	2.6	10175.0		

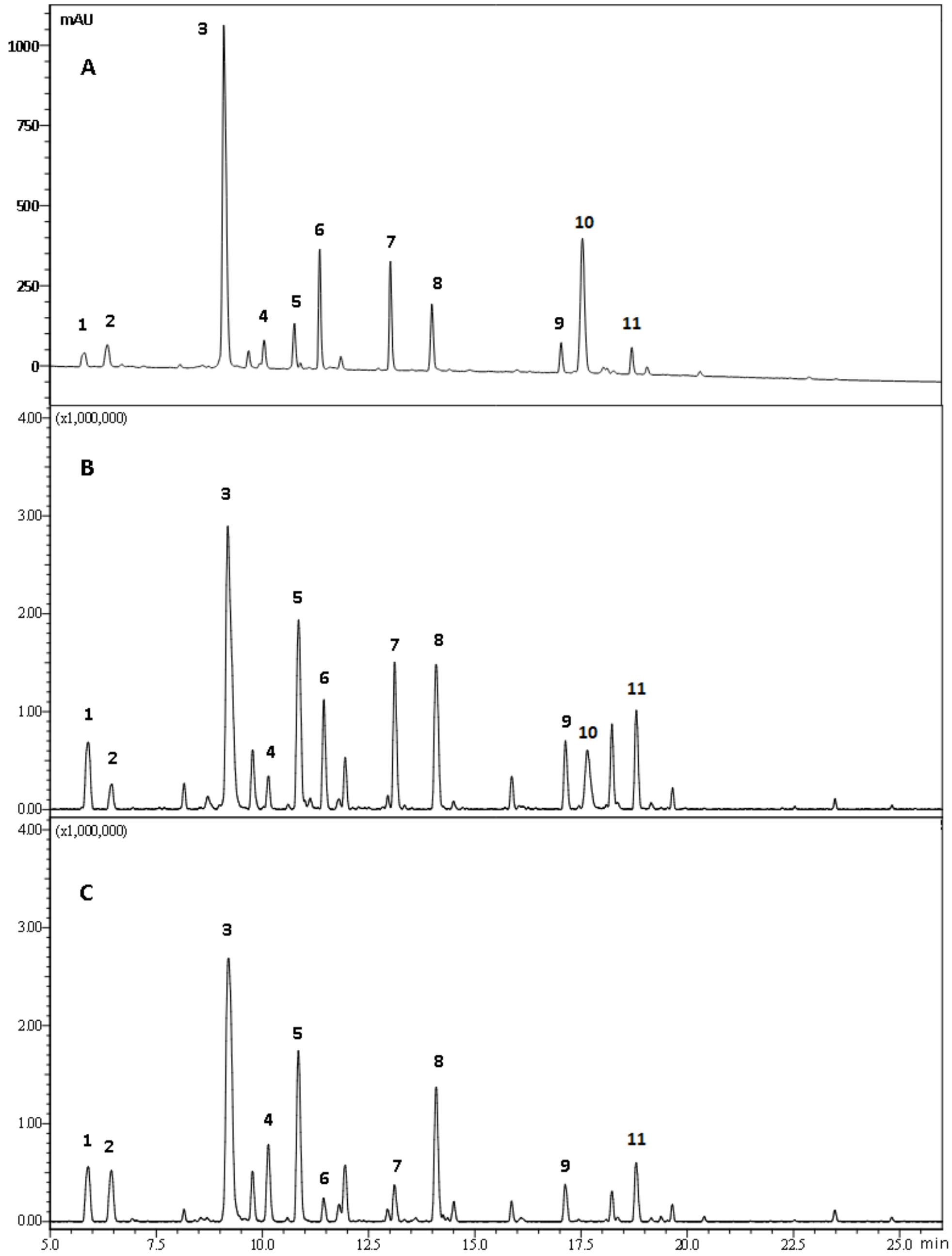

Figure S1. Representative chromatogram of *G. odoratum* (sun-grown) 70% ethanol extract, (A) UHPLC-PDA (254 nm), (B), and (C) UHPLC-HRMS in positive and negative ionization mode, respectively.

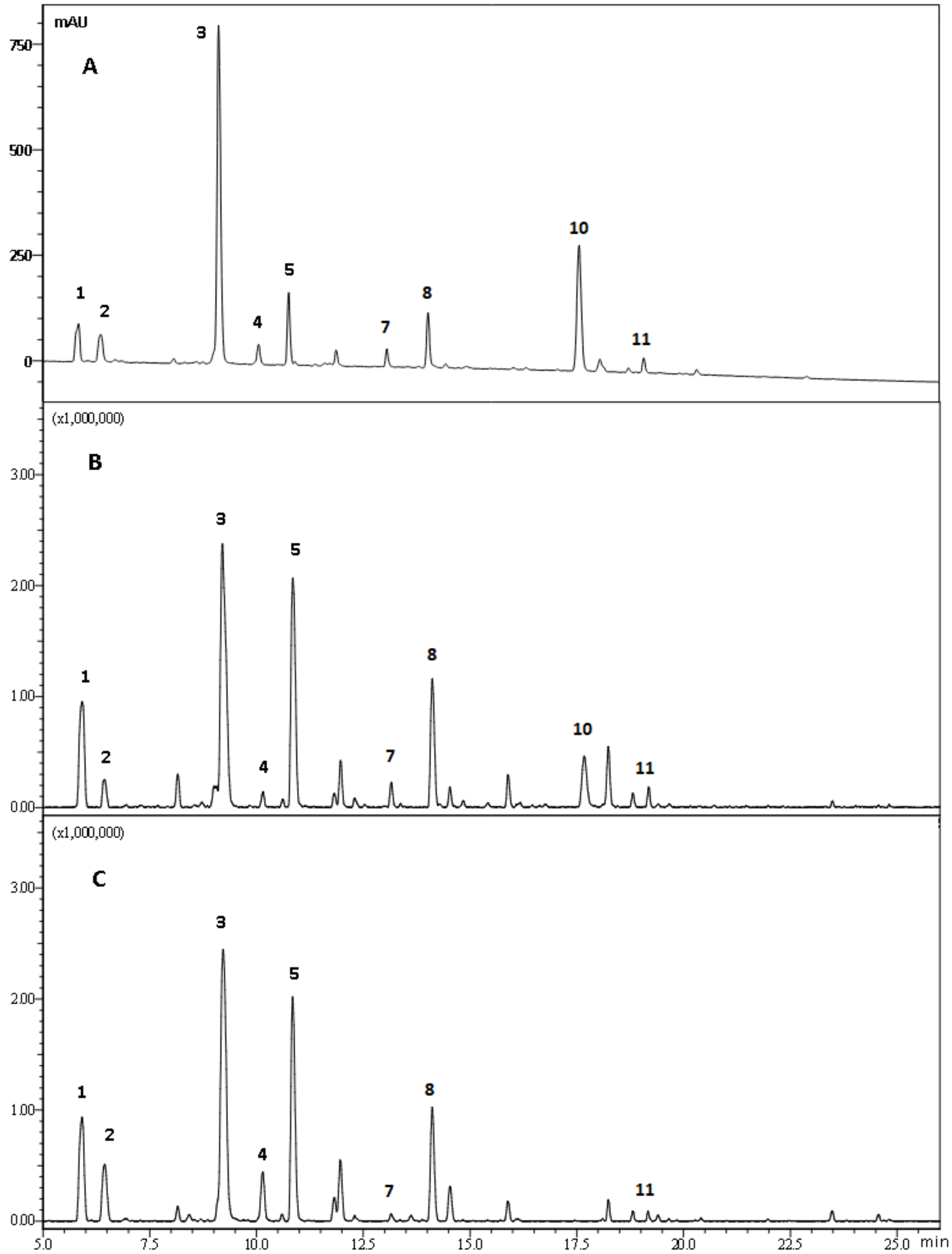

Figure S2. Representative chromatogram of *G. odoratum* (shade-grown) 70% ethanol extract, (A) UHPLC-PDA (254 nm), (B), and (C) UHPLC-HRMS in positive and negative ionization mode, respectively.

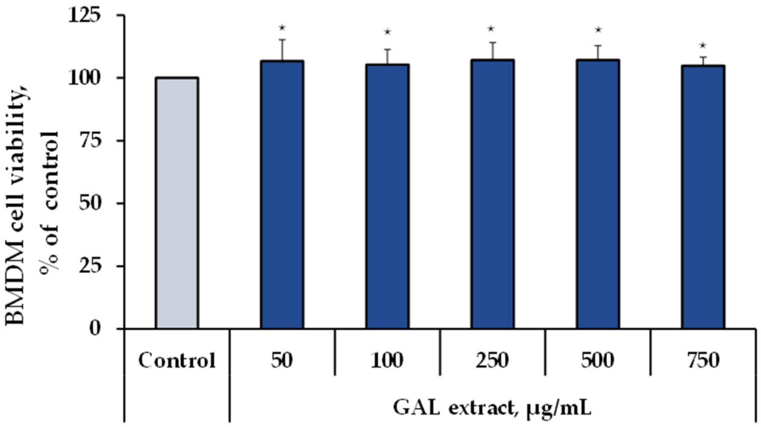

Figure S3. Effects of *G. odoratum* extract on bone marrow-derived macrophage viability measured by the MTT assay. Cell viability was tested after 24 h of incubation. Values are represented as the mean ± SD of three independent measurements in six parallels. * - significantly different from the untreated control (unpaired t-test, $p<0.05$).
